# RNA-binding protein CELF6 is cell cycle regulated and controls cancer cell proliferation by stabilizing p21

**DOI:** 10.1038/s41419-019-1927-0

**Published:** 2019-09-18

**Authors:** Gang Liu, Qianwen Zhang, Li Xia, Mengjuan Shi, Jin Cai, Haowei Zhang, Jia Li, Guanglan Lin, Weidong Xie, Yaou Zhang, Naihan Xu

**Affiliations:** 1 State Key Laboratory of Chemical Oncogenomics, Tsinghua Shenzhen International Graduate School, Tsinghua University, 518055 Shenzhen, China; 2 Key Lab in Healthy Science and Technology, Division of Life Science, Tsinghua Shenzhen International Graduate School, Tsinghua University, 518055 Shenzhen, China; 3 Open FIESTA Center, Tsinghua Shenzhen International Graduate School, Tsinghua University, 518055 Shenzhen, China

**Keywords:** Cell growth, Cell signalling

## Abstract

CELF6, a member of the CELF family of RNA-binding proteins, regulates muscle-specific alternative splicing and contributes to the pathogenesis of myotonic dystrophy (DM), however the role of CELF6 in cancer cell proliferation is less appreciated. Here, we show that the expression of CELF6 is cell cycle regulated. The cell cycle-dependent expression of CELF6 is mediated through the ubiquitin-proteasome pathway, SCF-β-TrCP recognizes a nonphospho motif in CELF6 and regulates its proteasomal degradation. Overexpression or depletion of CELF6 modulates p21 gene expression. CELF6 binds to the 3′UTR of p21 transcript and increases its mRNA stability. Depletion of CELF6 promotes cell cycle progression, cell proliferation and colony formation whereas overexpression of CELF6 induces G1 phase arrest. The effect of CELF6 on cell proliferation is p53 and/or p21 dependent. Collectively, these data demonstrate that CELF6 might be a potential tumor suppressor, CELF6 regulates cell proliferation and cell cycle progression via modulating p21 stability.

## Introduction

RNA-binding proteins (RBPs) play crucial role in posttranscriptional regulation in eukaryotes and control multiple aspects of cell behavior. RBPs often bind to mRNAs or noncoding RNAs to regulate all steps of RNA biogenesis, including pre-mRNA splicing, polyadenylation, transport and localization, mRNA stability, and translation^1–3^. RBPs bind to specific sequences or secondary structures of target RNAs through RNA-binding domains (RBDs). The most widely studied RBDs are RNA-recognition motif, K-hology domain, double stranded RNA-binding domain (dsRBD), and zinc finger. An RNA-binding protein contains one or more different RBDs, which allow high flexibility for interaction with different RNA targets^4^. Considering that RBPs coordinate the networks of protein-RNA and protein–protein interactions that control RNA metabolism, aberrant expression of RBPs have been implicated in many human diseases such as cancer^5^. RBPs control the expression of numerous oncogenes or tumor suppressors through posttranscriptional gene regulation. Many RBPs are deregulated in cancer and play critical roles in tumorigenesis and cancer development.

CUGBP Elav-like family (CELF) proteins are highly conserved based on their sequence-specific binding to RNA containing CUG repeats^6,7^. The human CELF family has six members, which can be divided into two subfamilies CELF1-2 and CELF3-6^8,9^. The mammalian CELF family proteins are broadly expressed in many tissues except that CELF3 and CELF5 are only found in the nervous system^9–11^. CELF protein are found in the nucleus and cytoplasm, the major role of nuclear CELF is regulating alternative splicing of target pre-mRNAs, the cytoplasmic CELF is implicated in the control of mRNA translation and/or stability. CELF proteins have been implicated in pathogenesis in various human diseases. CELF1 is upregulated in heart and skeletal muscle in myotonic dystrophy (DM) patients, disruption of CELF1-mediated alternative splicing is linked to DM symptoms^12–14^. Both CELF1 and CELF2 are upregulated in DM brains, transcripts that are dysregulated in the DM brain have been shown to be targets of CELF-mediated alternative splicing^15,16^. Emerging evidences suggest that CELF family proteins are central regulators in malignancies. A transposon-based genetic screen in mice identified CELF1 as a potent cancer driver in colorectal cancer^17^. Depletion of CELF1 results in growth inhibition, decreased cell viability, and apoptosis in mice and several cancer cells^18–22^. Conversely, overexpression of CELF1 prevents apoptosis in both HeLa and oral cancer cells^23,24^. Recent studies report that CELF1 regulates the translation of ten drivers of epithelial to mesenchymal transition (EMT), CELF1 is significantly overexpressed in human breast cancer tissues and is necessary and sufficient for both EMT and metastatic colonization^25^. On the contrary, CELF2 is a putative tumor suppressor in colon cancer, overexpression of CELF2 results in reduced colony formation and apoptosis by mitotic catastrophe in pancreatic and colon cancer cells^26–28^. CELF2 also plays a critical role in apoptosis in breast cancer cells in response to radiation injury^29^.

Many studies have focused on CELF1-2 subfamily, the function of CELF3-6 subfamily received much less attention. Mammalian CELF6 is expressed in many tissues, preferentially in kidney, brain, and testis^30^. CELF6 activates exon inclusion and exon skipping in a tissue-specific or developmental stage-specific manner. *Celf6−/−* mice exhibit a partial autism spectrum disorder-like phenotype, polymorphisms in the CELF6 gene may contribute to autism risk in human^31^. *Celf6*-YFP transgenic mice study shows that the gene products are present early in neurodevelopment and in adulthood, disruption of *Celf6* expression in hypothalamic nuclei may impact a variety of behaviors downstream of neuropeptide activity^32^. In this report, we aimed to study the function of CELF6 in cancer cell proliferation. We show that the expression of CELF6 is cell cycle regulated. The cell cycle-dependent expression of CELF6 is mediated through the ubiquitin-proteasome pathway, the E3 ubiquitin ligase SCF (SKP1-CUL1-F-box)-β-TrCP is responsible for CELF6 degradation. Gene expression profiling and KEGG pathway enrichment analysis reveal that the p53 signaling is enriched in *CELF6* knockout cells. Depletion or overexpression of CELF6 results in dramatic change of p21 expression. CELF6 binds to p21 mRNA and regulates its stability. CELF6 modulates cell cycle progression and cell proliferation in p53 and/or p21-dependent manner. Thus, we propose that CELF6 is a potential tumor suppressor, CELF6 regulates cancer cell proliferation and cell cycle progression via modulating p21 stability.

## Results

### The expression of CELF6 is cell cycle regulated

**Fig. 1:**
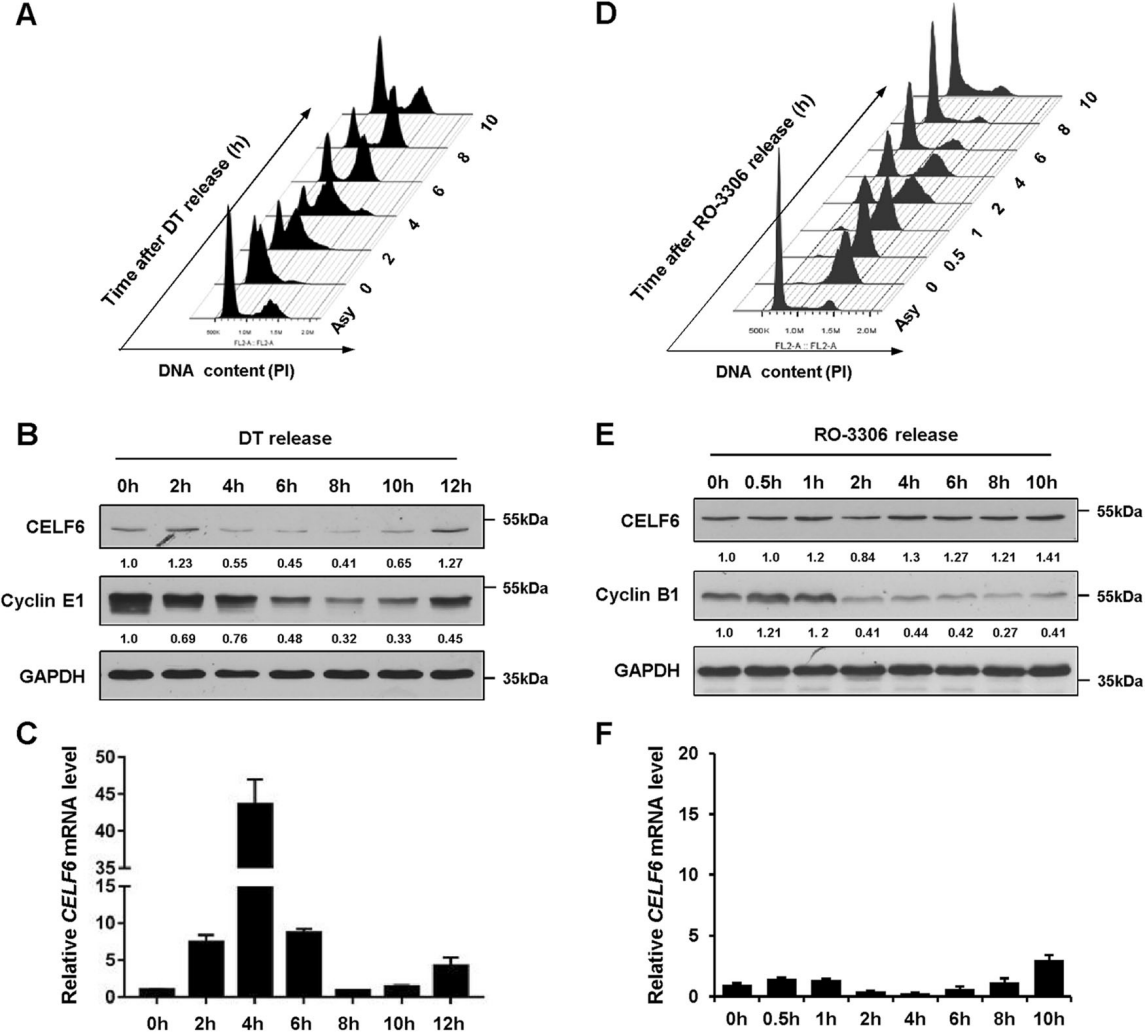
The expression of CELF6 is cell cycle regulated. **a** HCT116 cells were synchronized at the G1/S boundary by using double-thymidine (DT) block, cells were released from thymidine treatment at the indicated time points, fixed and stained with Propidium iodide (PI) for flow cytometry. **b** Cell extracts were collected at different time pointes after DT release and analyzed by immunoblotting, cyclin E1 was used as a G1/S phase protein marker. **c** Relative *CELF6* mRNA levels were determined by quantitative RT-PCR. **d** HCT116 cells were synchronized at the G2/M transition by CDK1 inhibitor RO-3306 treatment, cells were released from RO-3306 treatment at the indicated time points and cell cycle distribution was analyzed by flow cytometry. **e** Cell extracts were collected at different time pointes after RO-3306 release and analyzed by immunoblotting or **f** quantitative RT-PCR, cyclin B1 was used as a G2/M phase protein marker

To examine whether the expression of CELF6 is cell cycle regulated, the HCT116 colorectal cancer cells were synchronized at the G1/S boundary by a double-thymidine (DT) block, cells were released and harvested at different time points to perform flow cytometry and immunoblotting analysis. Immunoblotting revealed that CELF6 protein was relatively higher at G1/S and early S phases, then decreased sharply 4 h post DT release and maintained a relatively low level until most of the cells entered G2/M phase, following an increase in the amount of CELF6 at 10–12 h post DT release (G1 phase) (Fig. 1a, b). However, quantitative RT-PCR (qPCR) demonstrated that the expression patterns of CELF6 protein and mRNA are different, *CELF6* mRNA levels increased dramatically 4 h post DT release, indicating that posttranscriptional modifications may regulate the fluctuation of CELF6 protein during the cell cycle (Fig. 1c). Then, we used a selective CDK1 inhibitor RO-3306 to arrest cells at the G2/M phase border (Fig. 1d). The G2/M phase marker cyclin B1 was used as an indicator for immunoblotting of synchronized cell extracts. CELF6 mRNA and protein maintained at relatively constant levels during G2/M and early G1 phases, followed by accumulation of CELF6 protein in late G1 (Fig. 1e, f). We also analyzed CELF6 expression in HCT116 *p21−/−* cells, the protein level of CELF6 is still cell cycle regulated in *p21−/−* cells (Supplementary Fig. 1).

### CELF6 is degraded by the ubiquitin-proteasome pathway

**Fig. 2:**
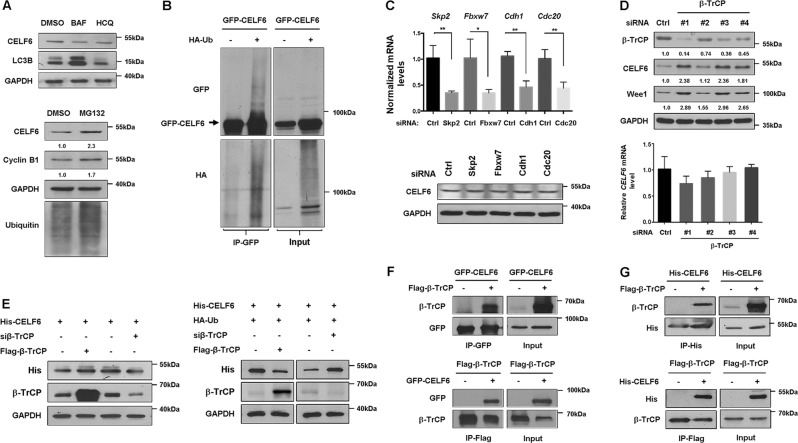
CELF6 is degraded by ubiquitin-dependent proteasome pathway. **a** HCT116 cells were treated with DMSO, 10 nM bafilomycin A1 (BAF) for 24 h, 20 μM hydroxychloroquine (HCQ) for 6 h, or MG132 (20 μM) for 6 h. The protein levels of CELF6, LC3B, cyclin B1, and Ubiquitin were analyzed by immunoblotting. **b** HEK293 cells were cotransfected with GFP-CELF6 and HA-Ub, cell extracts were subjected to immunoprecipitation with anti-GFP beads. The input and immunoprecipitates were analyzed by immunoblotting. **c** HCT116 cells were transfected with siRNAs against control, *Skp2*, *Fbxw7*, *Cdh1*, *Cdc20*, cell extracts were collected and subjected to immunoblotting analysis, RNAs were extracted for quantitative RT-PCR analysis to determine the silencing effect of each siRNA. **d** HCT116 cells were transfected with control and four different *β-TrCP* siRNAs, samples were analyzed by immunoblotting and quantitative RT-PCR analysis. **e** HCT116 cells were transfected with His-CELF6, Flag-β-TrCP or *β-TrCP* siRNA, and/or HA-Ub. The protein levels of His-CELF6 and β-TrCP were determined by immunoblotting. **f** HEK293 cells transfected with GFP-CELF6 and/or Flag-β-TrCP were subjected to immunoprecipitation with anti-GFP or anti-Flag antibody. The interaction between CELF6 and β-TrCP was determined by immunoblotting. **g** HEK293 cells transfected with His-CELF6 and/or Flag-β-TrCP were subjected to immunoprecipitation with anti-His or anti-Flag antibody. The input and immunoprecipitates were analyzed by immunoblotting

Both autophagy-lysosomal pathway and the ubiquitin-proteasome system control degradation of the majority of eukaryotic proteins^33^. To investigate which pathway contributes to CELF6 degradation, HCT116 cells were treated with the lysosomal inhibitor bafilomycin A1 (BAF) or hydroxychloroquine (HCQ), or the proteasome inhibitor MG132 before harvesting cells for immunoblotting. Both BAF and HCQ did not affect CELF6 expression, whereas the proteasomal inhibitor MG132 stabilized CELF6, indicating that CELF6 is degraded by proteasome pathway (Fig. 2a). The majority of proteins that are destroyed by proteasome are marked by the covalent attachment of polyubiquitin chains. To examine whether CELF6 is covalently modified through ubiquitination, HA-Ubiquitin and GFP-CELF6 were coexpressed in HEK293 cells, cell extracts were immunoprecipitated with GFP antibody. Several slow migrating bands were detected in cells cotransfected with GFP-CELF6 and HA-Ubiquitin, the bands can be detected by both GFP and HA antibodies suggesting that CELF6 is degraded by ubiquitin-conjugated proteasome pathway (Fig. 2b).

### Ubiquitination of CELF6 is mediated by SCF-β-TrCP

Two important E3 ubiquitin ligases, the anaphase-promoting complex (APC) and the SCF containing complex, are responsible for destroying the cell cycle regulators^34^. To explore the mechanism of CELF6 degradation during the cell cycle, we knocked down the expression of several F-box proteins (Skp2, Fbxw7, β-TrCP) or the APC activators (Cdc20, Cdh1). Immunoblotting revealed that CELF6 protein expression was not affected by *Skp2*, *Fbxw7*, *Cdc20*, or *Cdh1* siRNA (Fig. 2c). Interestingly, knockdown of *β-TrCP* significantly increased the protein level of CELF6 without affecting its mRNA expression. Wee1, a well-known β-TrCP substrate, was also accumulated in *β-TrCP* depleted cells (Fig. 2d). We also examined whether overexpression or silencing β-TrCP can regulate exogenous CELF6 degradation. HCT116 cells were transfected with His-CELF6, Flag-β-TrCP or β-TrCP siRNA, and/or HA-tagged ubiquitin. Immunoblotting for exogenous CELF6 indicated reduced levels of His-CELF6 in cells expressing Flag-β-TrCP and HA-Ub, whereas β-TrCP siRNA dramatically increased the level of His-CELF6 (Fig. 2e). Next, we investigated whether CELF6 can physically associate with β-TrCP. Co-immunoprecipitation results showed that Flag-β-TrCP or GFP-CELF6 was purified from cell extracts immunoprecipitated with GFP or Flag antibody (Fig. 2f). In addition, His-tagged CELF6 was also detected in Flag-β-TrCP immune complexes and Flag-β-TrCP was observed in His-CELF6 immunoprecipitates (Fig. 2g). These data indicate that CELF6 is a potential substrate of SCF-β-TrCP.

### SCF-β-TrCP regulates CELF6 degradation in a noncanonical manner

**Fig. 3:**
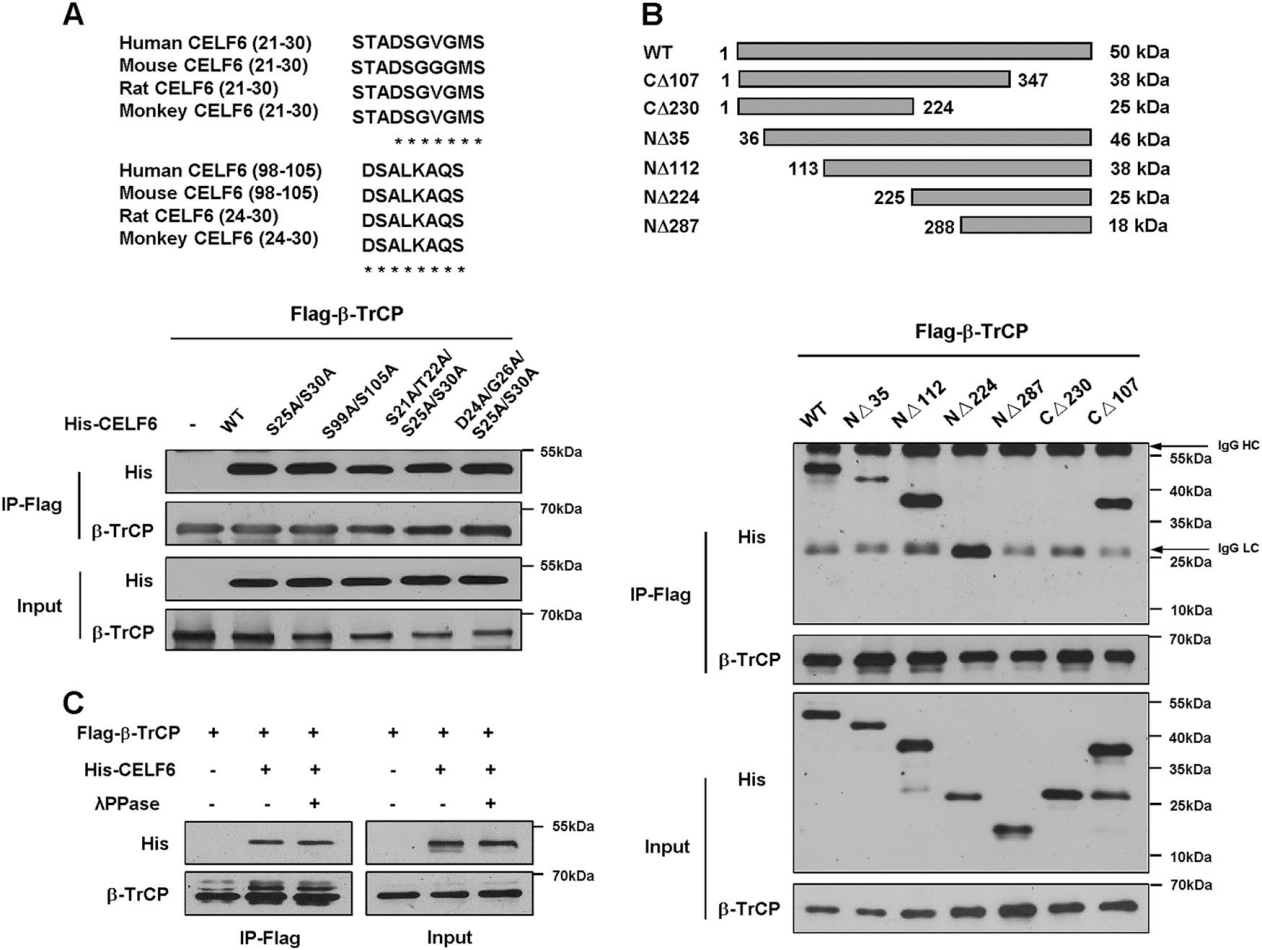
SCF-β-TrCP targets CELF6 for degradation. **a** Alignment of DSG(X_2-_n)S motif in CELF6 in different species. HEK293 cells were transfected with Flag-β-TrCP, wild-type His-CELF6 or point mutations, cell extracts were subjected to immunoprecipitation with anti-Flag beads. The interaction between CELF6 and β-TrCP was determined by immunoblotting. **b** Schematic of CELF6 and six truncated CELF6 fragments. Cell lysates from HEK293 cells transfected with Flag-β-TrCP and His-CELF6 truncations were subjected to immunoprecipitation with anti-Flag beads. The interaction between CELF6 and β-TrCP was determined by immunoblotting. Arrows indicate IgG heavy chain (HC) and light chain (LC). **c** HEK293 cells were transfected with Flag-β-TrCP and His-CELF6, cell extracts were treated with or without λPPase and subjected to immunoprecipitation and immunoblotting analysis

Most SCF-β-TrCP substrates have a consensus-binding motif DSG(X_2-_n)S and dual phosphorylation within the motif is important for high-affinity binding^35,36^. We analyzed the amino acid sequence of CELF6 and identified a conserved motif for β-TrCP recognition (the DSGVGMS motif). To test the importance of this motif, we generated two CELF6 point mutations, in one the two serines were changed to alanine (S25A/S30A), in another both the DSG sites and serine were changed to alanine (D24A/S25A/G26A/S30A). However, none of the mutant proteins affected the interaction between CELF6 and β-TrCP. We also identified another potential β-TrCP-binding motif (resides between amino acids 98 and 105), the two serine residues were mutated to alanine (S99A/S105A). Co-immunoprecipitation result showed that mutant proteins did not affect CELF6/β-TrCP association (Fig. 3a). Therefore, we propose that the DSG(X_2-_n)S motif is not required for β-TrCP recognition and binding.

To precisely identify the potential β-TrCP-binding sequence, we constructed several truncated mutations of CELF6 (NΔ35, NΔ112, NΔ224, NΔ287, CΔ107, and CΔ230) and examined their interaction with β-TrCP. The truncations were coexpressed with Flag-β-TrCP in HEK293 cells, co-immunoprecipitation results revealed that NΔ35, NΔ112, NΔ224, and CΔ107 interacted with β-TrCP whereas NΔ287 and CΔ230 did not bind to β-TrCP, indicating the β-TrCP binding site likely resides in the region between amino acids 224 and 288 in CELF6 protein (Fig. 3b). We also examined the subcellular localization of CELF6 mutants to rule out that the loss of interaction could be a consequence of β-TrCP and CELF6 localization in different cellular compartments. Immunofluorescent staining showed that all the CELF6 mutant proteins colocalized with β-TrCP (Supplementary Fig. 2).

Most substrates require phosphorylation to interact with SCF-β-TrCP, to test whether the interaction between CELF6 and β-TrCP is phosphorylation-dependent, we performed co-immunoprecipitation assay after λ phosphatase treatment. Surprisingly, the association between CELF6 and β-TrCP was not abolished by λ phosphatase (Fig. 3c). Therefore, our results suggest that β-TrCP binds to noncanonical degron in CELF6 and regulates its degradation in a phosphorylation-independent manner.

### Depletion of *CELF6* affects p53 signaling pathway

**Fig. 4:**
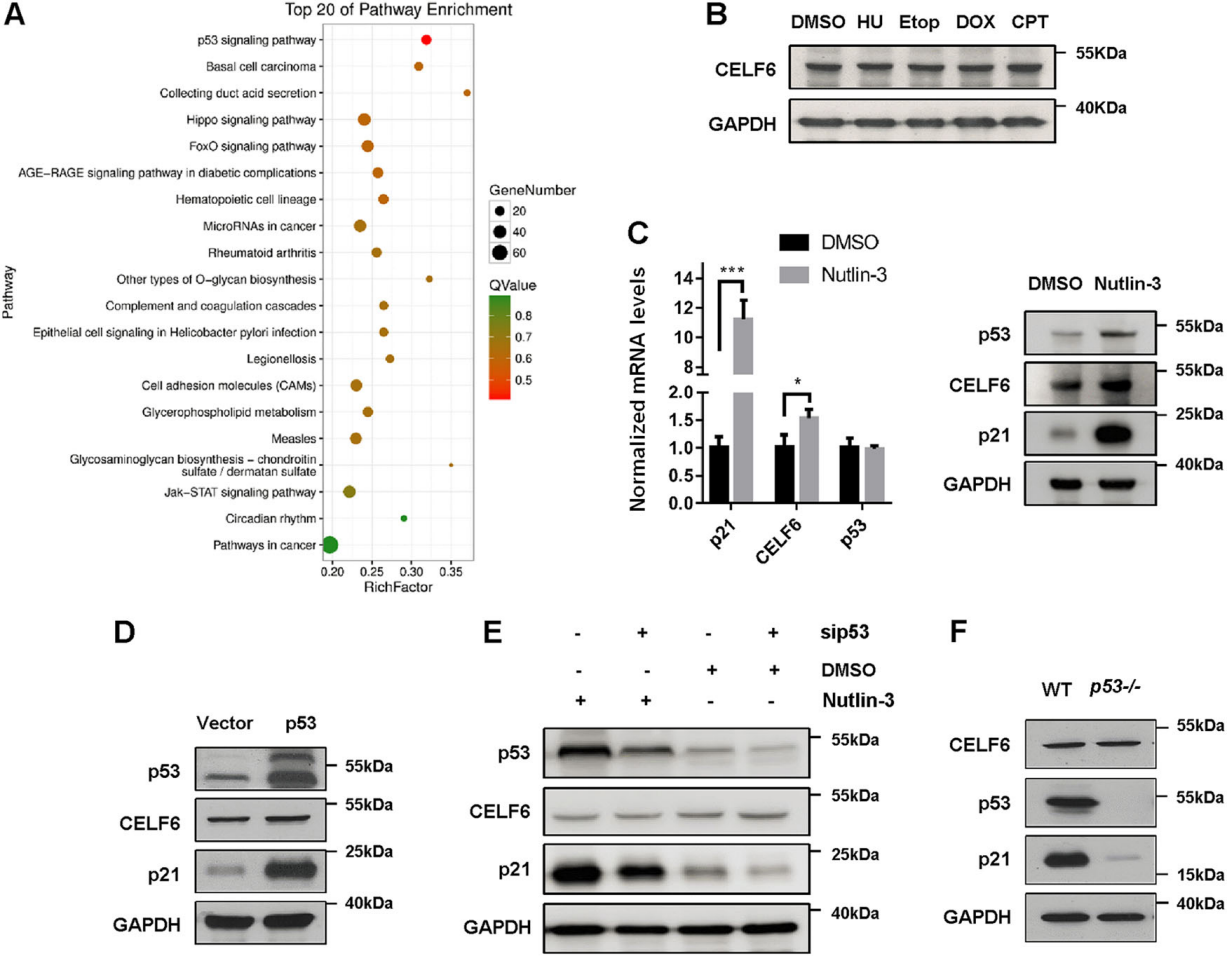
p53 signaling is enriched in *CELF6* knockout cells and p53 does not regulate CELF6 expression. **a** Control or *CELF6* knockout cells were performed transcriptome analysis. The differential gene expression in control and knockout cells were subjected to GO and KEGG pathway enrichment analysis, p53 signaling was the most enriched pathway in *CELF6* knockout cells. **b** HCT116 cells were treated with hydroxyurea (1 mM), etoposide (20 μM), doxorubicin (2 μM), or camptotechin (1 μM) for 12 h, the protein levels of CELF6 were analyzed by immunoblotting. **c** HCT116 were treated with or without nutlin-3 (20 μM) for 24 h, samples were collected for quantitative RT-PCR or immunoblotting to detect the expression levels of p21, CELF6, and p53. **d** HCT116 cells transfected with vector alone or p53-GFP were analyzed by immunoblotting. **e** HCT116 cells transfected with control or *p53* siRNA were treated with or without nutlin-3 for 24 h, cell lysates were analyzed by immunoblotting to detect the protein levels of p53, p21, and CELF6. **f** HCT116 wild-type (WT) and *p53*−/− cell lysates were analyzed by immunoblotting to determine the protein expression of CELF6, p53, and p21

To explore the function of CELF6 in cell proliferation, we used CRISPR/Cas9 technique to stably knock out *CELF6* in HCT116 cells. Transcriptome and gene expression analysis were performed to compare the differential expression levels of transcripts between control and *CELF6* knockout cells. The differentially expressed genes were subjected to enrichment analysis of GO functions and KEGG pathways. Several signaling pathways were enriched in *CELF6* knockout cells, among them, p53 signaling pathway was the most affected (Fig. 4a).

p53 is one of the most important tumor suppressor and transcription factor, therefore we determined whether the expression of CELF6 is controlled by p53. Many genotoxic agents can activate p53, we treated HCT116 cells with the DNA synthesis inhibitor hydroxyurea, the topoisomerase II inhibitor etoposide or doxorubicin, or the topoisomerase I inhibitor camptothecin for 12 h, none of the drugs modified the protein level of CELF6 (Fig. 4b). Nutlin-3, a nongenotoxic activator of p53, slightly increased *CELF6* mRNA level, but the protein expression of CELF6 was not induced by nutlin-3 (Fig. 4c). Consistently, overexpression or depletion of p53 did not affect CELF6 protein expression (Fig. 4d–f). These results suggest that the expression of CELF6 is not regulated by p53.

**Fig. 5:**
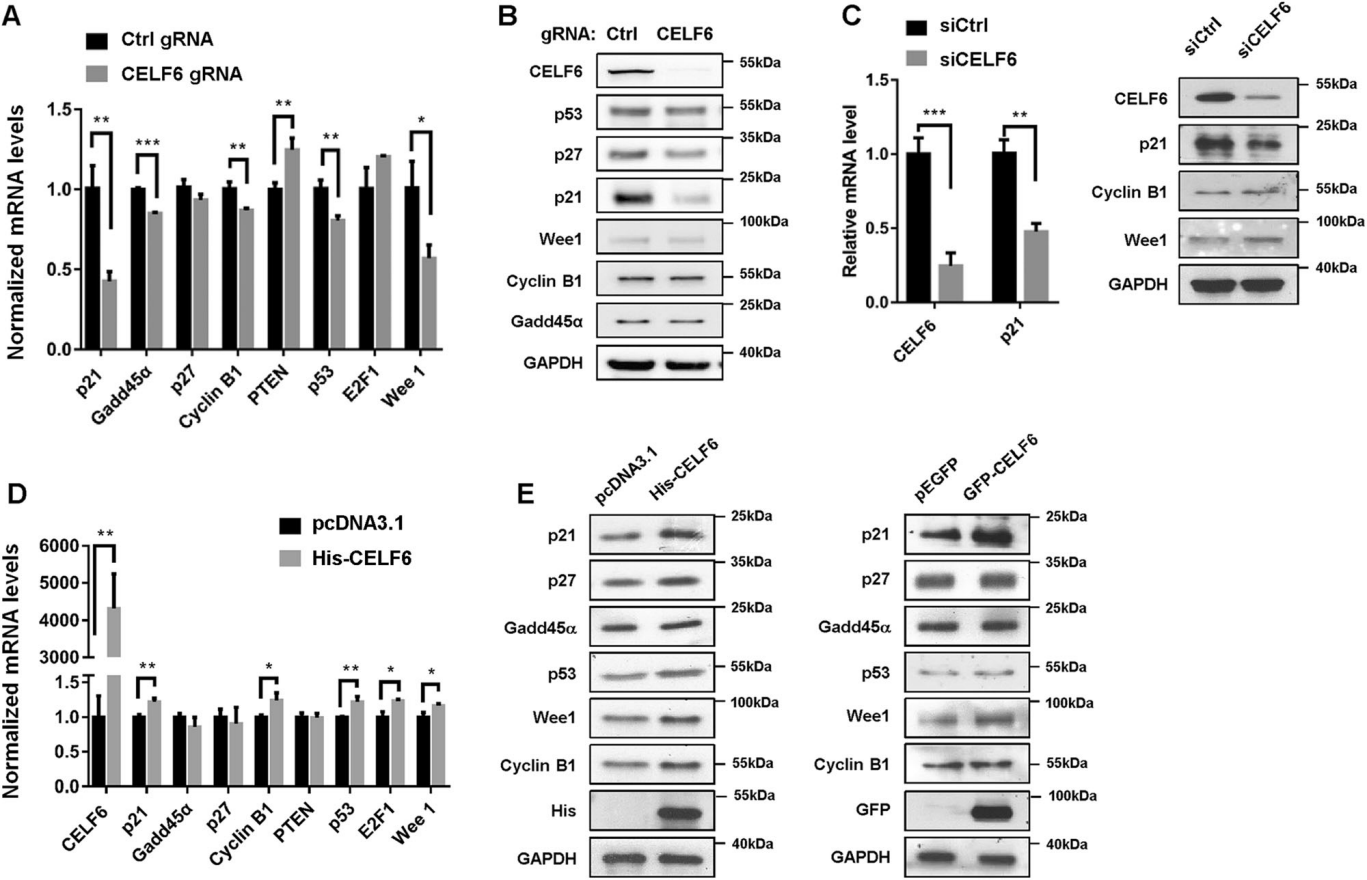
CELF6 modulates the expression of p21. **a** Detection of the mRNA expression levels of *p21*, *Gadd45α*, *p27*, *cyclin B1*, *PTEN*, *p53*, *E2F1*, and *Wee1* in control and *CELF6* knockout HCT116 cells. **b** The protein levels of p53, p21, p27, Wee1, cyclin B1, Gadd45α, and CELF6 were analyzed by immunoblotting. **c** Quantitative RT-PCR and western blot analysis in HCT116 cells transfected with control or *CELF6* siRNA. **d** HCT116 were transfected with pcDNA3.1 or His-CELF6 plasmids for 48 h, qPCR was performed to determine the expression of *p21*, *Gadd45α*, *p27*, *cyclin B1*, *PTEN*, *p53*, *E2F1*, *CELF6*, and *Wee1*. **e** HCT116 cells overexpressing His-CELF6 or GFP-CELF6 were subjected to immunoblotting analysis to determine the protein expression of p53, p21, p27, Wee1, cyclin B1, Gadd45α, and exogenous CELF6

Next we investigated whether CELF6 regulate the expression of p53 signaling. Quantitative RT-PCR assay revealed the expression of *p21*, *p53*, *Gadd45α*, *cyclin B1*, *PTEN*, and *Wee1* were modulated in *CELF6* knock out cells (Fig. 5a). However, immunoblotting showed that only p21 protein level was dramatically reduced by *CELF6* depletion (Fig. 5b). Then, we used a commercial siRNA pool to transiently knock down *CELF6* in HCT116 cells, the mRNA and protein expression of p21 were significantly downregulated by *CELF6* siRNA (Fig. 5c). We also knocked out *CELF6* expression in human hepatocellular carcinoma cell line HepG2, both qPCR and immunoblotting demonstrated that p21 expression was dramatically reduced in *CELF6* knock out HepG2 cells (Supplementary Fig. 3A, B). Exogenous CELF6 acted in opposite way on the regulation of *p21*, *p53*, *cyclin B1*, and *Wee1* transcripts, only p21 protein level was significantly increased in cells transfected with His- or GFP-tagged CELF6 (Fig. 5d, e).

### CELF6 regulates p21 mRNA stability

**Fig. 6:**
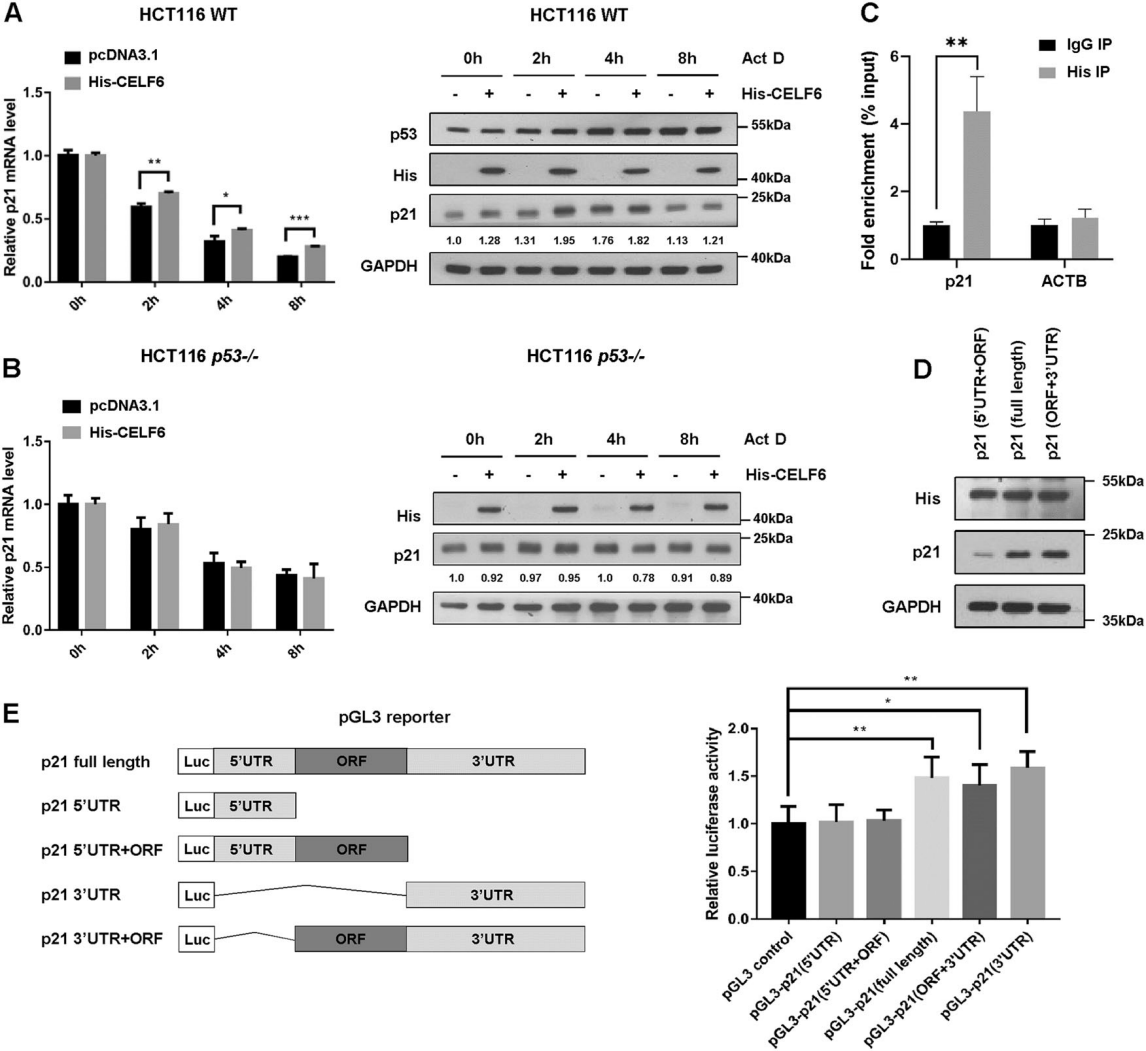
CELF6 regulates the stability of *p21* transcript. **a** HCT116 wild-type cells or **b** HCT116 *p53*−/− cells overexpressing empty vector or His-CELF6 were treated with actinomycin (Act D) at 5 μg/mL for different time points, samples were collected for quantitative PCR and immunoblotting analysis to detect the expression levels of p21. **c** HCT116 cells overexpressing His-CELF6 were lysed, the cell lysates were immunoprecipitated using either anti-His antibody or control IgG. CELF6-associated *p21* RNA was validated by qPCR using specific primers (***p < 0.001). *ACTB* was used as a negative control. **d** HCT116 cells were transfected with His-CELF6 and p21 expression vector containing 5′UTR + ORF, 3′UTR + ORF, or 5′UTR + 3′UTR + ORF (full length), the expression level of p21 was determined by immunoblotting. **e** Schematic diagrams or pGL3 reporters containing different combinations of *p21* transcript. HCT116 cells were cotransfected with various pGL3 reporter, pRL-TK and His-CELF6, luciferase activity was determined using a dual-luciferase reporter kit

RBPs play a major role in posttranscriptional control of RNAs, including mRNA stability. To test whether CELF6 is involved in regulating *p21* mRNA stability, we treated cells with the transcription inhibitor actinomycin D to inhibit de novo RNA synthesis. *p21* mRNA was gradually reduced in control cells treated with actinomycin D, overexpression of His-CELF6 delayed *p21* mRNA decay, indicating that CELF6 is able to stabilize the *p21* transcript. Actinomycin D has been shown to stabilize and activate p53, immunoblotting revealed that actinomycin D induced protein expression of p53 and its transcriptional target p21, ectopic expression of CELF6 further increased p21 protein level (Fig. 6a). We performed the same experiments in HCT116 *p53−/−* cells, overexpression of His-CELF6 did not affect *p21* mRNA stability and protein expression, indicating the effect of CELF6 on p21 expression is p53-dependent (Fig. 6b).

Then, we performed RNA immunoprecipitation (RIP) and qPCR to determine if CELF6 physically associates with *p21* mRNA. HCT116 cells were transfected with His-CELF6, the potential CELF6-RNA complex was immunoprecipitated with anti-His antibody. CELF6-bound mRNAs were purified for cDNA synthesis and quantified by qPCR with specific primers. *p21* transcripts were found to associate with His-CELF6, but the negative control *ACTB* mRNA was not precipitated from the His-IPs (Fig. 6c). To determine whether the 5′UTR or the 3′UTR is necessary for CELF6 to induce p21 expression, we generated expression vector containing p21 protein coding region (ORF) in combination with either the 3′UTR, the 5′UTR, or both. Immunoblotting showed that deletion of p21 3′UTR dramatically reduced the protein level of p21, indicating that 3′UTR is necessary for CELF6 mediated induction of p21 (Fig. 6d). We also performed a luciferase assaying using pGL3 reporter that contains various regions of p21 transcript (full length, 5′UTR, 5′UTR + ORF, 3′UTR, 3′UTR + ORF). Upon cotransfection with His-CELF6, a significant increase of luciferase activity was observed in cells expressed reporters containing 3′UTR (Fig. 6e). Collectively, these data suggest that CELF6 may regulate p21 mRNA stability through its 3′UTR region.

### CELF6 regulates cell cycle progression and cell proliferation

**Fig. 7:**
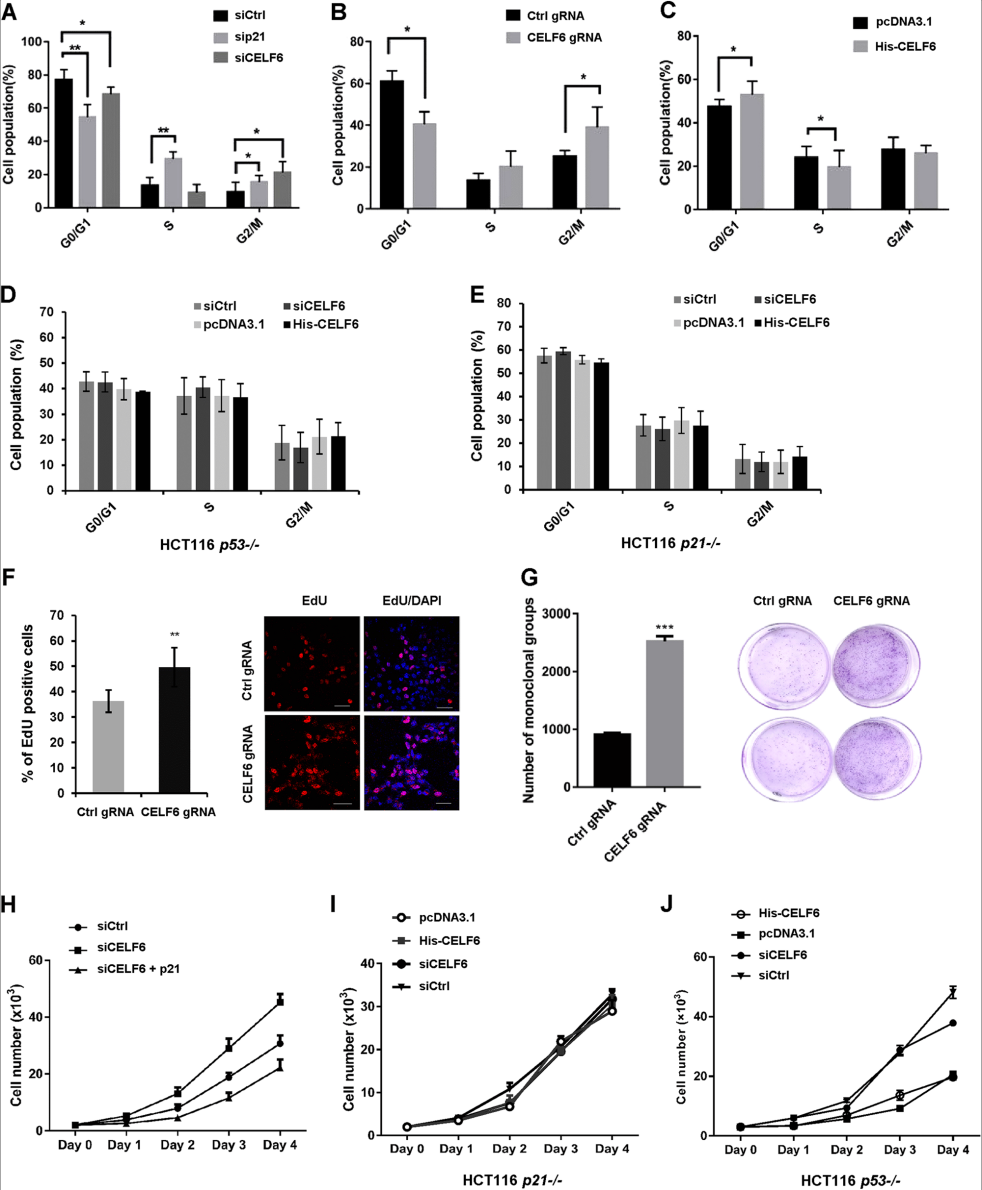
CELF6 modulates cell cycle progression and cell proliferation. **a** HCT116 cells transfected with control, *p21*, or *CELF6* siRNA for 48 h, the cell cycle distribution was analyzed by flow cytometry. **b** Control or *CELF6* knockout HCT116 cells were fixed and stained with PI, the cell cycle distribution was analyzed by flow cytometry. **c** HCT116 cells overexpressing empty vector or His-CELF6 were fixed, the percentage of G0/G1, S, and G2 phase cells was determined by flow cytometry. **d** HCT116 *p53*−/− or **e** HCT116 *p21*−/− cells were transfected with control or *CELF6* siRNA, pcDNA3.1, or His-CELF6 for 48 h, the cell cycle distribution was analyzed by flow cytometry. **f** Control or *CELF6* knockout cells were labeled with EdU for 30 min, cells were fixed and performed Click-iT EdU Alexa Fluor 555 imaging assay. The percentage of EdU-positive cells was determined by counting at least 300 cells for each sample. Scale bar, 50 μm. **g** Colony formation assay in control and *CELF6* knockout HCT116 cells. **h** CCK8 cell proliferation assay in HCT116 cells transfected with control siRNA, *CELF6* siRNA, or *CELF6* siRNA plus p21 plasmid. **i** CCK8 cell proliferation assay in HCT116 *p21*−/− or **j** HCT116 *p53*−/− cells transfected with control or *CELF6* siRNA, pcDNA3.1, or His-CELF6

To determine the physiological function of CELF6 in cell proliferation, HCT116 cells were transfected with control, *p21*, or *CELF*6 siRNA for 48 h, flow cytometry was applied to detect the cell cycle distribution. Silencing or knockout *CELF6* significantly decreased the G0/G1 ratio and increased the number of S or G2/M phase cells, *p21* siRNA showed the same result (Fig. 7a, b). In contrast, overexpression of CELF6 exhibited increased number of G0/G1 phase cells and decreased number of S phase population (Fig. 7c). We examined the effect of CELF6 on cell cycle in HCT116 *p53−/−* and *p21−/−* cells, overexpression or silencing CELF6 did not affect cell cycle distribution in both cells (Fig. 7d, e). We also performed EdU cell proliferation assay, the percentage of S phase cells was determined by measuring EdU incorporation. As predicted, the population of EdU-positive cells was markedly increased in *CELF6* knockout cells (Fig. 7f). Then we performed colony formation assay and CCK8 cell proliferation assay. The number of colonies increased dramatically in *CELF6* knockout cells (Fig. 7g). Depletion of *CELF6* also showed an increased proliferation capacity relative to the control cells, overexpression of p21 reversed the effect of *CELF6* siRNA on cell proliferation (Fig. 7h, supplementary Fig. 3C). However, overexpression or the depletion of *CELF6* did not affect cell proliferation in HCT116 *p53−/−* and *p21−/−* cells (Fig. 7i, j). These results indicate that CELF6 plays inhibitory effect in cell cycle and cell proliferation and the effect is p53 and/or p21-dependent.

## Discussion

The involvement of CELF family proteins in regulating cell cycle progression is less appreciated. In this study, we show that CELF6 protein level fluctuates throughout the cell cycle, SCF-β-TrCP is responsible for ubiquitin-dependent proteolysis of CELF6. β-TrCP, a member of the F-box protein family, is the substrate recognition subunit of the SCF E3 ubiquitin ligase complex^33,35^. β-TrCP plays important role in regulating cell cycle progression, cell growth, and survival. In general, β-TrCP recognizes a conserved binding motif with the sequence of DpSGΦXpS where Φ is a hydrophobic amino acid^35,37^. Although there is a conserved DSGVGMS motif in the N-terminus of CELF6, mutation analysis suggests that this conserved motif is not recognized by β-TrCP. β-TrCP has the ability to bind to noncanonical phosphodegrons, even a nonphospho-motif can be recognized by β-TrCP^36,38–40^. We show here the interaction between CELF6 and β-TrCP is not affected by λ phosphatase treatment, indicating β-TrCP binds to CELF6 in a phosphorylation-independent manner. Truncation analysis identified the sequence between amino acids 224–287 is sufficient and necessary for β-TrCP association. The β-TrCP recognition site locates in the divergent domain of CELF6. The divergent domain likely plays a role in mediating the interaction with RNA, but it is not clear whether the divergent domain affects protein–proteins interactions. Our results thus provide new evidence that β-TrCP can bind to nonconsensus motif in a phosphorylation-independent manner.

The cyclin-dependent kinase inhibitor p21 is a well-known cell cycle inhibitor and a major target of p53. p21 plays multiple function in cell cycle progression, cellular senescence, apoptosis, and transcriptional regulation in response to a variety of stimuli^41^. The expression of p21 is tightly controlled at transcriptional and post-transcriptional levels^41–44^. There are several lines of evidence suggesting that many RBPs, including HuR, HuD, Rbm24, RNPC1, and poly(C)-binding proteins (PCBPs) regulate p21 mRNA stability through binding the 3′UTR of p21 transcript^45–50^. On the other hand, CELF family protein CELF1 induces p21 expression via binding to a GC-rich sequence in the 5′UTR of p21 mRNA, overexpression of CELF1 increases p21 protein expression without increasing the cellular amount of the mRNA^10,51^. In this study, we found that depletion of *CELF6* affected p53 signaling pathway in HCT116 colorectal cancer cells. Both quantitative PCR and immunoblotting analysis revealed that the expression of p21 was dramatically modified by CELF6. RIP-qPCR and luciferase reporter assay demonstrated that CELF6 could bind to p21 3′UTR mRNA and regulate mRNA stability. In addition, CELF6 had no major effect on p53 expression and p53 did not regulate the expression of CELF6.

CELF family proteins play different roles in tumorigenesis in colorectal cancer. CELF1 is a potent cancer driver whereas CELF2 is potential tumor suppressor. To determine the function of CELF6 in cancer, we analyzed TCGA RNA-seq data set in colorectal cancer and performed immunohistochemistry assay in normal and cancerous colon tissues. Both CELF6 mRNA and protein expression levels were significantly lower in colon cancer tissues than those in nontumor colon tissues (Supplementary Fig. 4). Cell proliferation assay and flow cytometry analysis revealed that the overexpression of CELF6 induced G1 phase arrest, depletion of CELF6 promoted G1/S transition and cancer cell proliferation, overexpression of p21 reversed the effect of *CELF6* siRNA on cell proliferation, indicating that CELF6 is a potent tumor suppressor in colorectal cancer, the inhibitory effect of CELF6 is likely mediated through p21. However, we found that overexpression or silencing CELF6 did not affect cell cycle distribution and cell proliferation in HCT116 *p53−/−* and *p21−/−* cells, suggesting that CELF6 modulates cell cycle progression and cell proliferation in p53 and/or p21-dependent manner.

## Materials and methods

### Cell culture and synchronization

Human colorectal carcinoma cell HCT116 and human embryonic kidney cell HEK293 were purchased from the American Type Culture Collection, both cells were cultured in the recommended medium supplemented with 10% fetal bovine serum (Gibco BRL) at a 37 °C incubator with 5% CO_2_. The CRISPR/Cas9 system was used to generate *CELF6* knockout HCT116 cells. The lenti-CRISPR carrying control or *CELF6* guide RNA was customized by Genechem (Shanghai, China). Knockout cells were selected using puromycin resistance.

To synchronize cells in late G2 phase, HCT116 were treated with RO-3306 at 10 uM for 20 h. Cells were washed three times in phosphate buffer saline (PBS) and released in fresh medium for different time points. To synchronize cells in G1/S boundary, HCT116 cells were treated with thymidine at 2 mM for 16 h, released in fresh medium for 8 h and treated with thymidine again for 16 h. Cells were washed three times in PBS and released in fresh medium for different time points.

### Transcriptome and bioinformatics analysis

Transcriptome analysis was performed by Gene Denovo Biotechnology Co. (Guangzhou, China). Briefly, total RNAs in control or *CELF6* knockout cells were extracted using Trizol reagent. Eukaryotic mRNA was enriched by Oligo (dT) beads and reversed transcribed into cDNA with random primers. The cDNA fragments were purified, end-repaired, poly A added, and ligated to Illumina sequencing adapters. The ligation products were sequenced using Illumina HiSeq^TM^ 2500. Differently expressed genes (DEGs) were identified with a fold change ≥2 and a false discovery rate (FDR) <0.05 in a comparison as significant DEGs, DEGs were then subjected to enrichment analysis of GO functions and KEGG pathways. The Q value is an adjusted *p* value after multihypothesis test correction, taking FDR ≤0.05 as a threshold. The Richfactor refers to the ratio of the number of the differentially expressed to the total number of annotated genes, the larger the Richfactor value, the greater the degree of enrichment.

### Reagents

Transfection reagents Lipofectamine 2000 and Lipofectamine 3000 were purchased from Invitrogen, RNA synthesis inhibitor actinomycin D, protease inhibitor MG132, lysosomal inhibitors bafilomycin and hydroxychloroquine, CDK inhibitor RO-3306, and thymidine were purchased from Sigma-Aldrich. λPPase was purchased from New England BioLabs. Anti-CELF6 (ab173282) and anti-GFP (ab1218) were obtained from Abcam, anti-p21^Waf1/Cip1^ (#2947), anti-p27^Kip1^(#3686), anti-Gadd45α (#4632), anti-Cyclin B1(#12231), anti-β-TrCP (#4394), anti-Wee1 (#13084), anti-cleaved PARP (#5625), anti-cleaved Casp3 (#9661), anti-cyclin E1 (#20808), and anti-Ubiquitin (#3936) antibodies were purchased from Cell Signaling Technology, anti-P53 (10442-1-AP), anti-GFP (66002-1-1 g), anti-His (66005-1-1 g), anti-β-tubulin (60008-1-1 g), and anti-GAPDH (10494-1-AP) were obtained from Proteintech, anti-LC3B (L7543) was obtained from Sigma-Aldrich.

### Plasmids and siRNAs

His-CELF6 plasmid was synthesized by GenePharma (Shanghai, China). To generate GFP-CELF6, His-CELF6 was used as template, and the cloning primers were designed according to the sequence of human CELF6 gene (NCBI Gene ID: 60677). Flag-β-TrCP (#10865) and HA-Ubiquitin (#17608) were purchased from Addgene. All His-CELF6 truncation plasmids were generated by YouBio (Changsha, China). His-CELF6 point mutations were generated by Q5 Site-directed mutagenesis kit (E0552S, New England Biolabs) using His-CELF6 as the templates.

CELF6 siRNA (sc-90308), p21 siRNA (sc-29427), and p53 siRNA (sc-29435) were purchased from Santa Cruz Biotechnology. β-TrCP specific siRNAs were synthesized by RiboBio or purchased from Dharmacon. siRNA sequences were as follows: Skp2 oligo, 5′- GUACAGCACAUGGACCUAUTT-3′; Fbxw7 oligo, 5′- GCCUCCAGGAAUGGCUAAATT-3′; Cdh1 oligo, 5′- UGAGAAGUCUCCCAGUCAGUTT-3′; Cdc20 oligo, 5′- GGAGCUCAUCUCAGGCCAUTT-3′, which were synthesized by GenePharma.

### Immunoblotting and immunoprecipitation

Cells were lysed in ice-cold lysis buffer as described previously^52^. Cells extracts were mixed with sample loading buffer, heated and separated by SDS-PAGE and transferred to nitrocellulose membranes. The membranes were blocked with 5% nonfat milk and incubated with the corresponding primary antibodies and HRP-conjugated secondary antibodies. Protein bands were visualized by chemiluminescence (Thermo Fisher Scientific) and exposed to X-ray films.

For immunoprecipitation, cells were lysed in lysis buffer (20 mM Tris,150 mM NaCl, 1Mm EDTA 1 mM EGTA, 1% NP-40). Cell lysates were incubated with protein G (Invitrogen) binding antibodies for 1 h. the IP beads were washed by PBS/T three times, the co-immunoprecipitated proteins were eluted by heating the IP beads and analyzed by SDS-PAGE and immunoblotting.

### Quantitative RT-PCR

Total RNA was extracted using Trizol reagent following the manufacturer’s protocol (TaKara). Reverse transcription was performed using RT master mix (Toyobo), and PCR was run using SYBR Green (Toyobo). Primers used to amplify *CELF6*, *p53*, *p27*, *p21*, *Gadd45α*, *Wee1*, *PTEN*, *E2F1*, *cyclin B1*, and *ACTB* transcripts were listed in Table 1. *ACTB* was used as an internal control.

**Table 1:** Primers used for RT-PCR and RIP assay

Primer name	Sequence
ACTB-RT-F	5′-CATGTACGTTGCTATCCAGGC-3′
ACTB-RT-R	5′-CTCCTTAATGTCACGCACGAT-3′
CELF6-RT-F	5′-CCCATCGGGGTCAATGGATTC-3′
CELF6-RT-R	5′-GCCCGTTATTGTAGAGCGTGT-3′
Cyclin B1-RT-F	5′-AATAAGGCGAAGATCAACATGGC-3′
Cyclin B1-RT-R	5′-TTTGTTACCAATGTCCCCAAGAG-3′
p27-RT-F	5′-ATCACAAACCCCTAGAGGGCA-3′
p27-RT-R	5′-GGGTCTGTAGTAGAACTCGGG-3′
Gadd45α-RT-F	5′-GAGAGCAGAAGACCGAAAGGA-3′
Gadd45α-RT-R	5′-CAGTGATCGTGCGCTGACT-3′
Wee1-RT-F	5′-GACGAAGATGATTGGGCATCC-3′
Wee1-RT-R	5′-TGGACTGGAGATCCTTGTTACA-3′
PTEN-RT-F	5′-AGGGACGAACTGGTGTAATGA-3′
PTEN-RT-R	5′-CTGGTCCTTACTTCCCCATAGAA-3′
p53-RT-F	5′-AAAGTGCGTCCGTTCTCAATG-3′
p53-RT-R	5′-GGTTCTTCCTCAGAGTACCAAAG-3′
E2F1-RT-F	5′-CATCCCAGGAGGTCACTTCTG-3′
E2F1-RT-R	5′-GACAACAGCGGTTCTTGCTC-3′
p21-RT-F	5′-AGCGATGGAACTTCGACTTTG-3′
p21-RT-R	5′-CGAAGTCACCCTCCAGTGGT-3′

### Immunofluorescence and immunohistochemistry

HCT116 cells were co-transfected with Flag-β-TrCP and His-CELF6 mutants for 48 h, cells were fixed in 4% paraformaldehyde for 15 min and permeabilized with 0.5% Triton X-100 in PBS for 5 min. Fixed samples were blocked with 3% BSA/PBS, followed by incubation with anti-His (Proteintech, 66005-1-Ig) and Alexa Fluor 488 conjugated secondary antibody (Invitrogen). The stained samples were then incubated with anti-Flag (Proteintech, 66008-3-Ig) and Alexa Fluor 555 conjugated secondary antibody (Invitrogen). Images were captured using an Olympus FV1000 confocal microscope (Olympus, Japan).

For immunohistochemistry, paraffin-embedded cancerous colon tissues (*n* = 30) and normal colon tissues (*n* = 4) were purchased from Shanghai Outdo Biotech Co Ltd. The paraffin-embedded slide was deparaffinized, rehydrated, and blocked in sheep serum for 30 min, followed by incubation with anti-CELF6 (1:100, Abcam, ab173282) overnight at 4 °C. The slide was mounted with D.P.X. for histology analysis.

### RNA immunoprecititation (RIP)

HCT116 cells were transfected with His-CELF6 plasmid for 48 h, cells were lysed according to the instruction of RIP Kit (Sigma-Aldrich), then incubated with 1 μg of anti-His or isotype control IgG at 4 °C overnight. The RNA-protein immunocomplexes were pulled down by protein G beads and RT-PCR was performed to detect the expression of target RNA.

### EdU staining

Control or *CELF6* knockout HCT116 cells were labeled with 10 μM EdU for 30 min, then stained using a Click-iT Edu Alexa Fluor 555 Imaging Kit (Invitrogen). Images of EdU and DAPI were taken with an Olympus confocal microscope (FV1000, Olympus). EdU-positive cells were calculated from 300 cells in each sample.

### Luciferase assay

The pGL3 luciferase reporters containing p21 protein coding region (ORF) in combination with either the 3′UTR, the 5′UTR, or both were constructed by YouBio (Changsha, China). A dual luciferase assay was performed according to the manufacturer’s instructions (Promega). HCT116 cells were co-transfected with pGl3 reporter, pRL-TK, and His-CELF6 for 48 h. Samples were lysed, luciferase activity was measured using a dual-luciferase reporter kit. The data depicted are representative of three independent experiments.

### Flow cytometry analysis

HCT116 cells transfected with control, *p21* or *CELF6* siRNA, control or CELF gRNA, pcDNA3.1 or His-CELF6, HCT116 *p53*−/− or *p21*−/− cells transfected with control or CELF6 siRNA, pcDNA3.1 or His-CELF6 were fixed in 70% ethanol in PBS overnight. Cells were washed and resuspended in PBS, stained with propidium iodide (Sigma, P4170) and analyzed for DNA content by use of a BD Accuri C6 flow cytometer.

### CCK8 and colony formation assay

HCT116 wild-type, *p53*−/− or *p21*−/− cells transfected with control or *CELF6* siRNA, pcDNA3.1 or His-CELF6 were seeded into 96-well plate (3 × 10^3^ cells/well) and cultured for 24, 48, 72, and 96 h. Cells were treated with 10 μL CCK8 solution and incubated for 2 h at 37 °C. The absorbance of each well was quantified at 450 nm using a microplate reader (Thermo Fisher Scientific). The growth curve was obtained by calculating the number of cells based on a standard curve.

For colony formation assay, control or *CELF6* knockout HCT116 cells were seeded into 6-cm culture dishes (2 × 10^3^ cells/well) and cultured for 10 days. Cells were fixed with 4% paraformaldehyde and subsequently stained with crystal violet for 30 min. The number of colonies were counted in each dish.

### Statistical analysis

All data were expressed as mean ± s.d. of three independent experiments. Measurement data between normal and tumor tissues were performed using nonparametric Mann–Whitney test. Statistical analyses were performed using two-tailed Student’s *t*-test.

## Supplementary information

Supplementary Figure legend.

Supplementary Figure 1.

Supplementary Figure 2.

Supplementary Figure 3.

Supplementary Figure 4.
