# Supplementary material for: RNA-binding protein CELF6 is cell cycle regulated and controls cancer cell proliferation by stabilizing p21
Source: Cell Death Dis. 2019 Sep 18;10(10):688. doi: 10.1038/s41419-019-1927-0 (PMC6751195; doi:10.1038/s41419-019-1927-0)
Supplement: Supplementary file 1 — Supplementary Figure legend. [file 41419_2019_1927_MOESM1_ESM.docx]

**Supplementary data**

**Figure legends**

**Figure 1. The expression of CELF6 is cell cycle regulated in HCT116 *p21-/-* cells.** HCT116 *p21-/-* cells were synchronized at the G1/S boundary by double-thymidine (DT) block or at the G2/M transition by CDK1 inhibitor RO-3306. Cells were released from the treatment at the indicated time points, cell extracts were analyzed by immunoblotting.

**Figure 2. The subcellular localization of β-TrCP and CELF6 mutants.** HCT116 cells were co-transfected with Flag-β-TrCP and His-CELF6 WT or mutants for 48 h. Cells were fixed and stained with anti-Flag and anti-His antibodies. The fluorescent images were acquired using a confocal microscope. Scale bar, 10 μm.

**Figure 3. Depletion of CELF6 downregulates p21 and accelerates cell proliferation in HepG2 cells.** (A) Detection of the mRNA expression levels of *p21*, *Gadd45α*, *p27*, *cyclin B1*, *PTEN*, *p53*, *E2F1*, and *Wee1* in control and *CELF6* knockout HepG2 cells. (B) The protein levels of p53, p21, p27, Wee1, cyclin B1, Gadd45α and CELF6 were analyzed by immunoblotting. (C) CCK8 cell proliferation assay in control and *CELF6* knockout HepG2 cells.

**Figure 4. CELF6 is downregulated in colon cancer tissues.** (A) The TCGA colon cancer RNA-seq data set was analyzed, including 480 tumor tissues and 41 normal tissues. The expression level of CELF6 in normal tissues was significantly higher than that in tumor tissues (*^***^p*<0.001). (B) Immunohistochemical detection of CELF6 expression level in colon cancer tissues and normal tissues. (C) Relative CELF6 antibody staining intensity was evaluated by a semiquantitative approach based on a predominant staining intensity. The protein level of CELF6 in normal colon tissues (n=4) was significantly higher than that of tumor tissues (n=30) (*^*^p*<0.05).
