# Supplementary figures and images for: RNA-binding protein CELF6 is cell cycle regulated and controls cancer cell proliferation by stabilizing p21
Source: Cell Death Dis. 2019 Sep 18;10(10):688. doi: 10.1038/s41419-019-1927-0 (PMC6751195; doi:10.1038/s41419-019-1927-0)

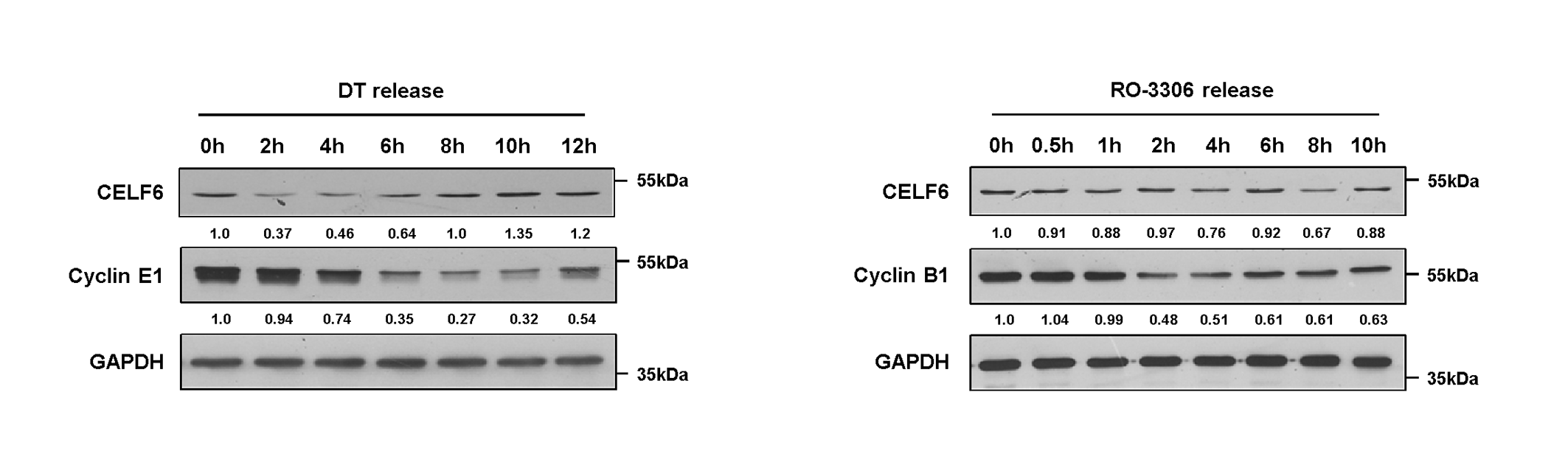

Supplement: Supplementary file 2 — Supplementary Figure 1. [file 41419_2019_1927_MOESM2_ESM.tif]

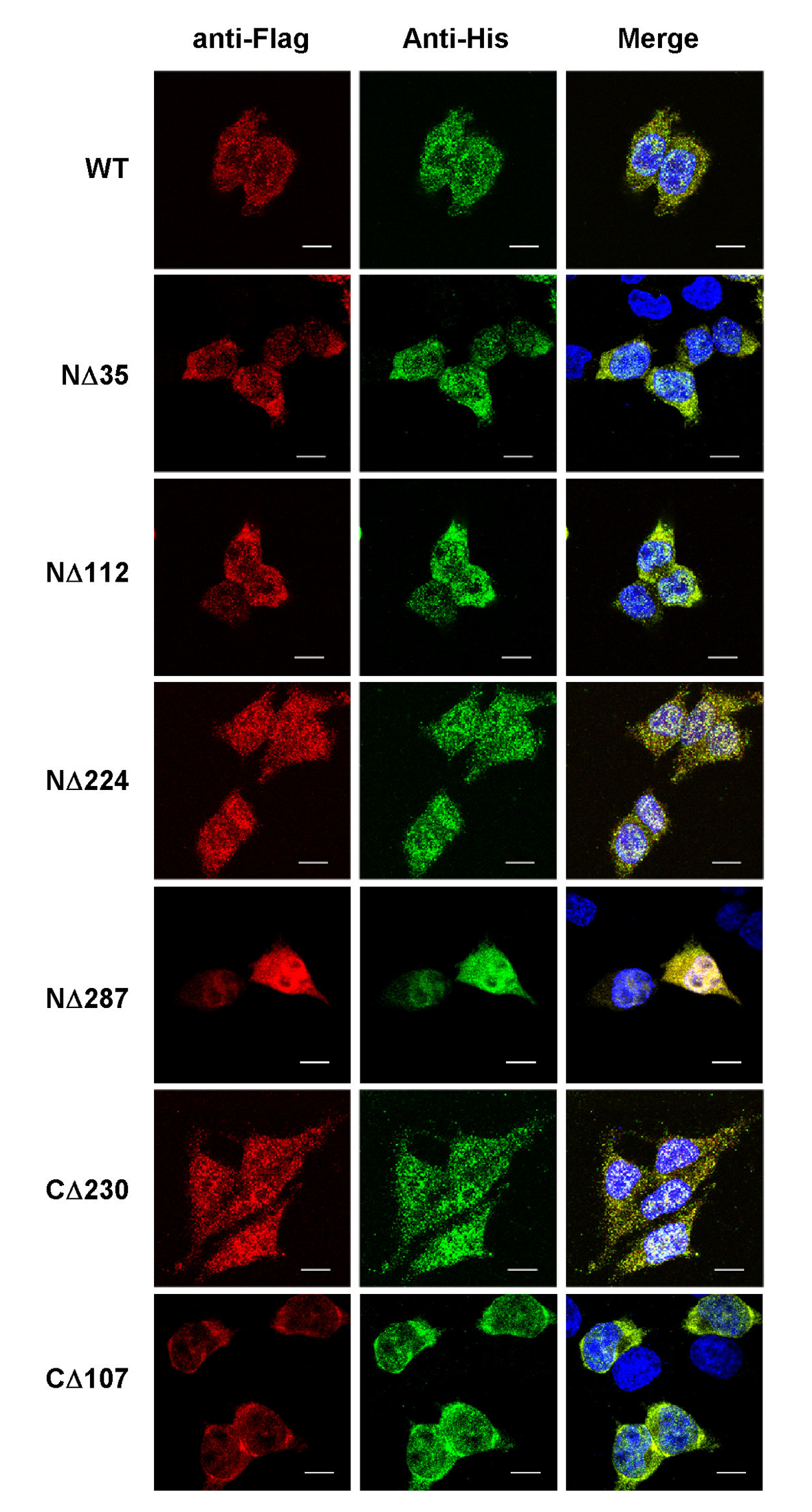

Supplement: Supplementary file 3 — Supplementary Figure 2. [file 41419_2019_1927_MOESM3_ESM.tif]

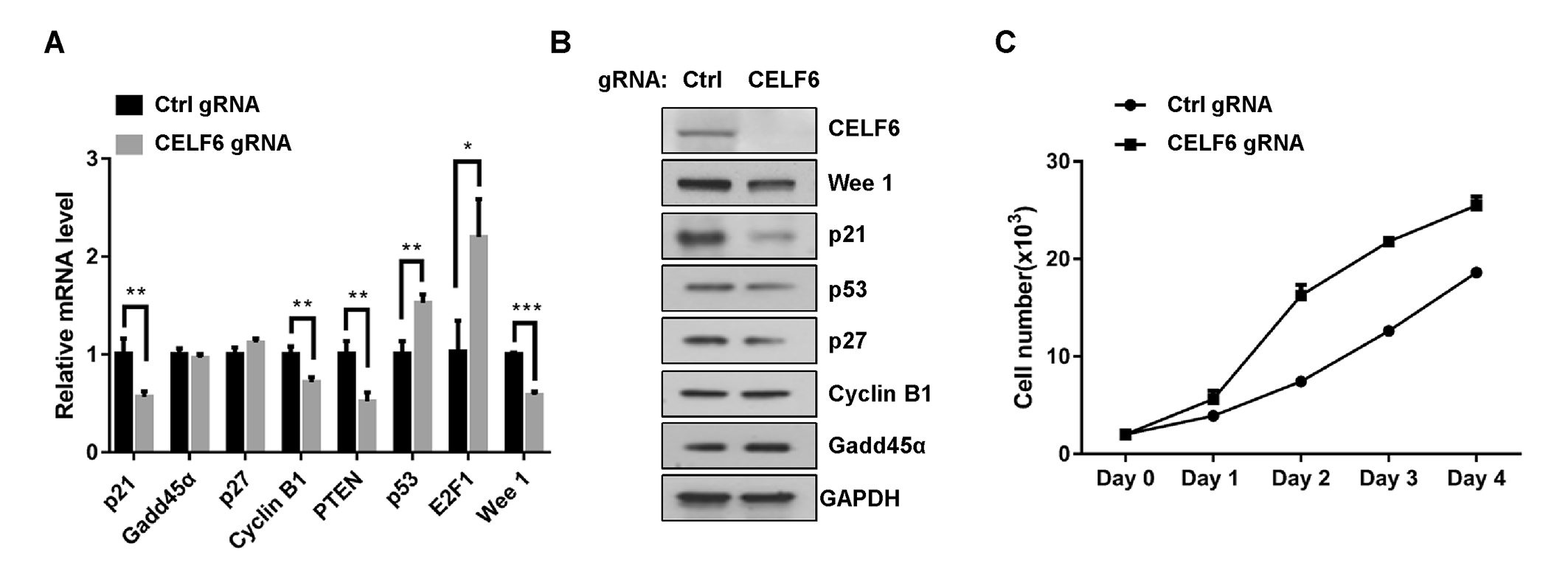

Supplement: Supplementary file 4 — Supplementary Figure 3. [file 41419_2019_1927_MOESM4_ESM.tif]

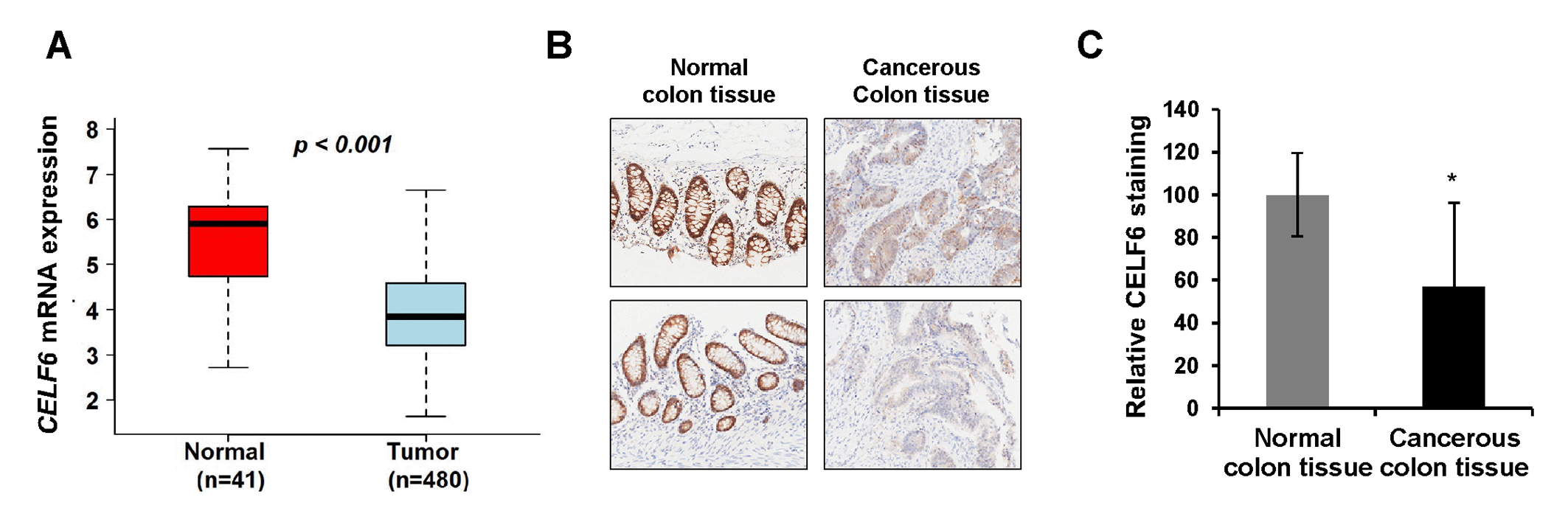

Supplement: Supplementary file 5 — Supplementary Figure 4. [file 41419_2019_1927_MOESM5_ESM.tif]
